# Perceptions of Undergraduate Medical Students on Group Effectiveness in Problem-Based Learning (PBL) at Qassim University

**DOI:** 10.7759/cureus.92831

**Published:** 2025-09-21

**Authors:** Mawahib Abu Elgasim, Wafaa Alotaibi, Lobaina Abozaid, Ramaze Elhakeem, Shimaa Soliman

**Affiliations:** 1 Department of Family and Community Medicine, College of Medicine, Qassim University, Buraidah, SAU; 2 Department of Medical Education, College of Medicine, Qassim University, Buraidah, SAU; 3 Department of Pathology, College of Medicine, Qassim University, Buraidah, SAU; 4 Department of Physiology, College of Medicine, Qassim University, Buraidah, SAU

**Keywords:** group effectiveness, medical education, problem-based learning, qassim university, saudi arabia, student perception

## Abstract

Background and objective

Group effectiveness in problem-based learning (PBL) plays a vital role in achieving learning outcomes; however, limited research has examined how students perceive this effectiveness, particularly in the Saudi educational context. This study aimed to explore undergraduate medical students’ perceptions of group effectiveness in PBL.

Methods

A cross-sectional survey was conducted among medical students using a validated questionnaire with a 5-point Likert scale. Descriptive statistics were used to analyze frequency and mean responses. Inferential analyses, including ANOVA and Tukey’s honestly significant difference post hoc tests, were performed to examine the impact of gender and academic level on perceptions.

Results

The majority of students reported positive perceptions of group effectiveness. Within the process construct, 125 (58.7%) preferred group learning over working alone, although concerns about time efficiency were noted. Within the content construct, 166 (77.7%) of students agreed that group work enhanced tolerance, collaboration, and peer learning. ANOVA revealed statistically significant differences by gender and academic level in both constructs (p < 0.05), with third-year students reporting more favorable perceptions than first-year students.

Conclusions

Students generally perceived PBL group work as effective, particularly in fostering collaborative skills, tolerance, and peer learning. However, concerns about time investment and participation dynamics remain.

## Introduction

Problem-based learning (PBL) is a student-centered educational strategy that encourages active engagement with real-world problems to develop critical thinking, self-directed learning, and collaborative skills [1]. Originally introduced in medical education, PBL has been globally recognized for its potential to improve clinical reasoning and foster lifelong learning habits [2,3]. The core structure of PBL involves small groups of students collaboratively analyzing clinical scenarios under the guidance of a facilitator, rather than relying on traditional didactic teaching [4].

In Saudi Arabia, the implementation of PBL in undergraduate medical education has significantly expanded over the past two decades, reflecting a broader commitment to align medical curricula with international standards and enhance training quality [5]. Several institutions, including Najran, Majmaah, Jouf, and Qassim universities, have integrated PBL into their medical programs, each adapting the approach to fit institutional resources and objectives [6-8]. Almulhem and Almulhem [9] conducted a cross-sectional study evaluating PBL implementation in a Saudi medical college, reporting generally positive student perceptions but highlighting the need for faculty development and adequate support systems to sustain effective group learning. Additionally, Roh and Kim [10] demonstrated that integrating PBL with simulation techniques significantly improves students’ motivation and life skills, supporting the notion that blended instructional methods can amplify the benefits of PBL.

These initiatives reflect increasing awareness of the value of PBL in strengthening students’ analytical reasoning, motivation, and communication competencies. Nevertheless, the effectiveness of PBL remains the subject of ongoing academic scrutiny, particularly concerning students’ perceptions of group collaboration and the quality of interaction within tutorial sessions. Systematic reviews and meta-analyses have identified group functionality and the overall educational environment as critical factors influencing PBL outcomes [11,12].

The implementation of PBL in the Middle East, especially within Saudi Arabia, is shaped by distinct cultural, institutional, and social dynamics that influence how students engage with peers and facilitators in group settings [13,14]. For instance, a recent scoping review by Trullàs et al. [15] revealed substantial variability in the effectiveness of PBL methodologies, often depending on the quality of student engagement within groups. Lim [16] argued that for sustainable implementation, institutions must address faculty and student concerns regarding unclear role expectations, assessment methods, and facilitator training.

Peer collaboration, professionalism, and the quality of group discussions are essential for maximizing the educational value of PBL tutorials [17]. PBL relies heavily on effective group collaboration, as collaborative skills are crucial for constructing shared understanding and solving complex problems. For example, Chen et al. [18] identified that students often struggle with time and task management but gradually develop self-regulatory strategies that enhance autonomous learning. Similarly, Smith et al. [19] reviewed self-management interventions and emphasized their potential to mitigate disruptive behaviors, thereby improving group productivity in educational settings.

Wang et al. [20] further explored how intercultural sensitivity and group ethnic composition affect PBL group dynamics, noting that diversity can enrich learning but also requires effective facilitation to navigate cultural differences productively. These findings underscore the importance of group composition and facilitator preparedness in supporting successful PBL implementation.

Notably, the College of Medicine, Qassim University (COMQU) was among the first institutions in Saudi Arabia to adopt a fully integrated PBL curriculum [12], positioning itself as a national model for innovative medical education. Evaluating student perceptions of group effectiveness within such programs is vital for ongoing curriculum refinement, particularly in light of regional cultural contexts and evolving pedagogical demands.

This study is expected to assist curriculum developers in enhancing teaching quality and sustaining Qassim University’s leadership in innovative education. It also aligns with Vision 2030’s goal of raising higher education quality and supports evidence-based teaching reforms in medical colleges throughout the Kingdom. Saudi Arabia’s Vision 2030 is a national strategic plan to enhance the quality of life and modernize government services by the year 2030. Launched in 2016, it has driven major social and economic reforms, with implementation structured in phases, the final phase commencing in 2026. A key connection to medical education is healthcare excellence: Vision 2030 aims to provide high-quality healthcare, and medical education trains doctors to meet these standards through modern teaching methods and evidence-based practice.

By addressing a local knowledge gap and providing context-specific insights, this study contributes to broader international discussions on culturally adaptable and effective PBL strategies.

This study aimed to measure medical students’ perceptions of the effectiveness of small-group PBL tutorials.

## Materials and methods

Study design

This was a cross-sectional, descriptive, institution-based study designed to explore the perceptions of undergraduate medical students regarding group effectiveness in PBL sessions.

Study area

The research was conducted at the COMQU, Saudi Arabia, a leading medical institution in the region. The College of Medicine was established in the academic year 2000-2001, and its curriculum follows the Student-centered, Problem-based, Integrated, Community-based, Elective, and Systematic (SPICES) model, which is recognized for its innovation and student engagement. The medical program spans six years and is divided into three structured phases. Phase I, the Preparatory Year, provides foundational sciences and essential academic skills. Phase II, the systems-based curriculum, is delivered during the first to third academic years and integrates clinical and basic sciences through PBL and modular teaching. Phase III, Clinical Clerkships, begins in the fourth year, during which students participate in practical, community, and hospital-based training under professional supervision.

Study population

The study targeted undergraduate medical students enrolled during the academic year 2020-2021 at COMQU. The population included both male and female students across three academic levels: first year (level 1), second year (level 2), and third year (level 3). These groups represent students who were actively engaged in PBL sessions and had experienced multiple cycles of group-based learning activities, making them well-suited to evaluate group effectiveness.

Study selection criteria

All undergraduate students in their first, second, and third academic years who were enrolled in the College of Medicine at the main campus of Qassim University were eligible. Students who discontinued their studies, dropped out during the academic year, or were absent from the majority of PBL sessions were excluded.

Sampling technique

To ensure fair representation of the population, proportional stratified sampling was used based on both academic level and gender. The total population consisted of 435 undergraduate medical students, distributed across three academic levels: 150 students in the first year, 151 in the second year, and 134 in the third year. The target sample size was 213 students, representing approximately 48.97% of the total population (213 ÷ 435 ≈ 0.4897).

The number of students sampled from each subgroup was calculated using the formula:

\(\text{Sample size for a subgroup} = \left( \frac{\text{Number of students in subgroup}}{\text{Total population}} \right) \times \text{Total sample size}\)

For the first year, the total number of students was 150, and the sample size was calculated as (150 ÷ 435) × 213 = 0.3448 × 213 ≈ 73.45, resulting in 73 students. For the second year, the total number of students was 151, and the sample size was (151 ÷ 435) × 213 = 0.3471 × 213 ≈ 73.95, resulting in 74 students. For the third year, the total number of students was 134, and the sample size was (134 ÷ 435) × 213 = 0.3080 × 213 ≈ 65.58, resulting in 66 students.

Table 1 presents the proportional distribution of sampled students derived from the total population of 435 across academic levels and gender groups.

**Table 1:** Distribution of undergraduate medical students and sample proportion (2020-2021)

Level	Male	Sample size	Female	Sample size	Total	Total sample size
First year	93	44	57	27	150	71
Second year	95	45	56	26	151	71
Third year	76	36	58	27	134	63
Total	264	125	171	80	435	205

After determining the required sample size for each subgroup, systematic random sampling was applied within each stratum to select participants. This approach ensured that the final sample accurately represented the overall student population while maintaining proportionality across academic levels.

Sample size

The sample size was determined using the formula:

$n = \frac{N}{1 + N \times D^2,}$

where n = sample size, N = population size (435), and D = degree of precision (0.05).

Applying this formula:

$n = \frac{450}{1 + 435 \times 0.0025} = 205$

Although the calculated sample size was 205 students, a total of 213 students participated in the study. This slight increase of approximately 4% was included to account for potential non-respondents and to ensure sufficient data for analysis.

Data collection tools and procedures

Data were collected using a validated, self-administered online questionnaire [21]. The questionnaire was distributed via Google Forms (Google LLC, Menlo Park, CA, USA) in compliance with COVID-19 health and safety protocols. It consisted of three main sections: demographic characteristics, process construct variables, and content construct variables (Appendix A).

The instrument was pretested on five students, who were excluded from the final sample. Their feedback was used to revise ambiguous items and improve clarity, thereby enhancing reliability and face validity.

Data analysis

Data were exported from Google Forms (Google LLC) and initially screened and cleaned in Microsoft Excel. Final analyses were conducted using IBM SPSS Statistics for Windows, Version 25.0 (Released 2017; IBM Corp., Armonk, NY, USA).

Likert scale responses were recoded into three categories for analysis: Agree (Strongly Agree + Agree), Neutral, and Disagree (Disagree + Strongly Disagree). Descriptive statistics (frequencies, percentages, means, and SDs) summarized participant responses. Inferential statistics included one-way ANOVA to compare mean scores by gender and examine variations in perceptions across academic years. A p-value < 0.05 was considered statistically significant in all tests.

Ethical considerations

Ethical approval was obtained from the Subcommittee of Health Research Ethics at Qassim University (reference number: 19-14-01). The study adhered to the principles of the Declaration of Helsinki for research involving human subjects.

Informed consent was obtained electronically at the beginning of the questionnaire. Only students who consented were able to proceed. Participants were assured of anonymity, confidentiality, and voluntary participation. No personally identifying information was collected, and data were used solely for academic and research purposes.

## Results

Demographic characteristics

A total of 213 undergraduate medical students from Qassim University participated in the study. Participants ranged in age from 18 to 24 years, with a mean age of 20.82 years (SD = 3.471).

**Figure 1:**
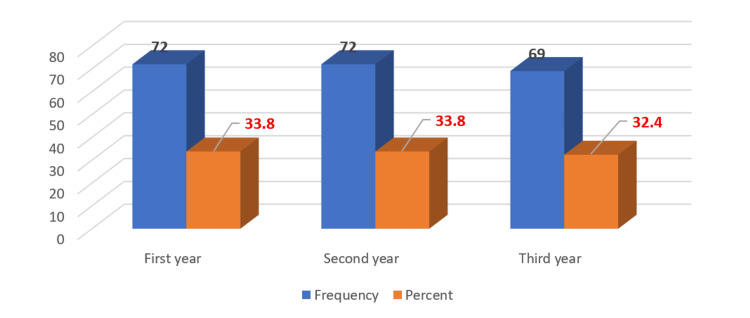
Distribution of participants across academic levels (n = 213)

Regarding gender distribution, 128 students (60.1%) were male and 85 students (39.9%) were female. With respect to academic level, participants were fairly evenly distributed across the first three years of the undergraduate medical program. Specifically, 72 students (33.8%) were enrolled in the first year, 72 (33.8%) in the second year, and 69 (32.4%) in the third year (Figure 1).

**Figure 2:**
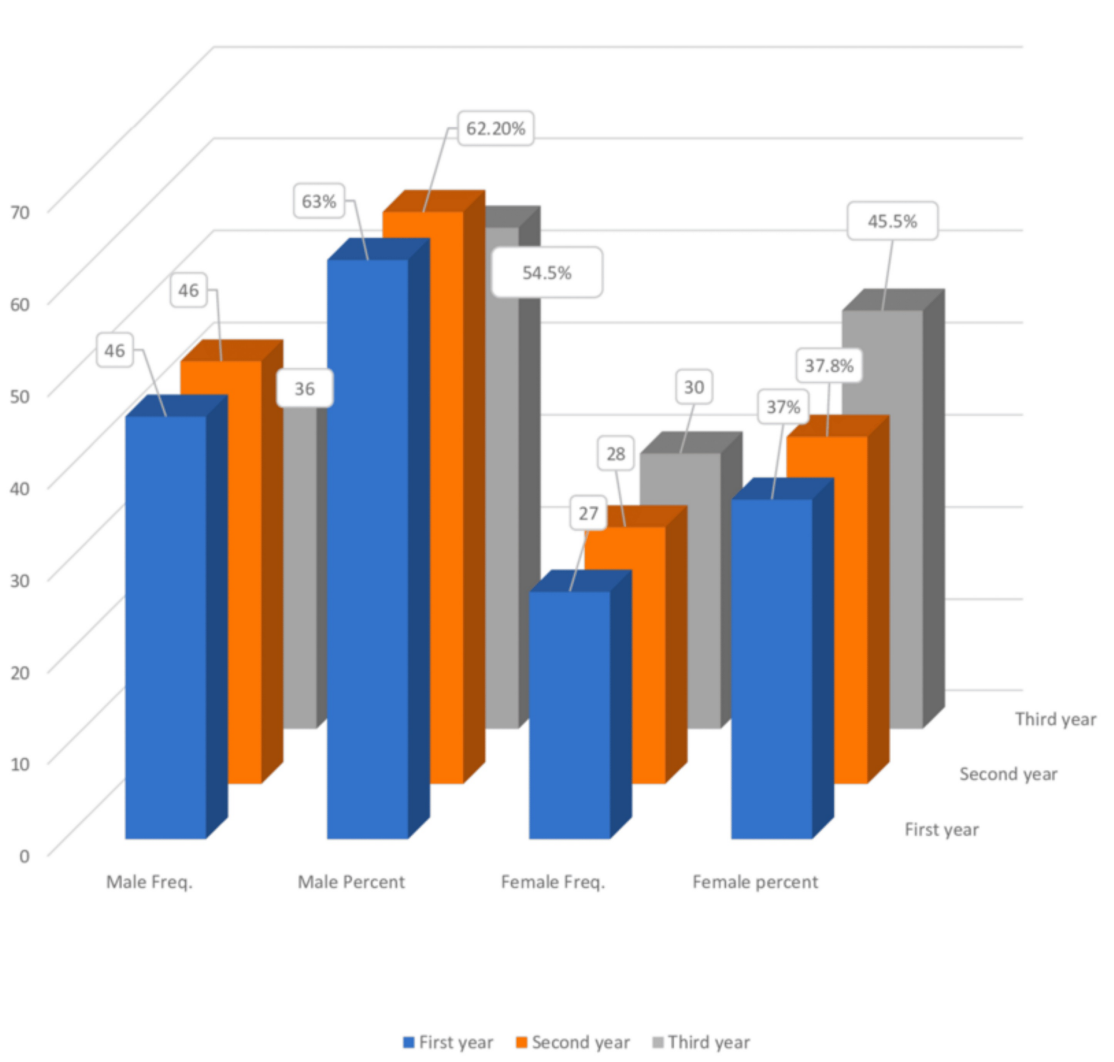
Cross-tabulation of gender and academic level

A cross-tabulation of gender and academic level showed that the second-year group included more males (45) than females (26). Similarly, the first-year group had more males (45) than females (27), while the third-year group consisted of 37 males and 32 females (Figure 2).

Perceptions of group effectiveness in the process construct

Table 2 presents the frequency distribution of students’ perceptions regarding the effectiveness of group work in PBL based on the process construct. A majority of students, 125 (58.7%), agreed that working in a group was better than working alone, indicating a strong preference for collaborative learning. Similarly, 80 students (37.4%) agreed that they learned less when working alone, although 55 (25.8%) disagreed, reflecting variation in individual learning preferences.

**Table 2:** Frequency distribution of perceptions of the effectiveness of group work in PBL (process construct) based on the Likert scale (n = 213) PBL, problem-based learning

Process construct	Agree	Neutral	Disagree
Working in a group was better than working alone	58.7%	24.9%	16.5%
When working alone, I did not learn as much as I did when I worked in a group	37.4%	26.8%	25.8%
Group learning did not take too much of my time	27.3%	26.8%	46%
Learning in a group was not frustrating and stressful	47.4%	28.6%	23.9%
Group learning was a very good way of learning the content of the block	56.3%	27.7%	16%

Regarding time efficiency, only 58 students (27.3%) agreed that group learning did not take too much time, while 98 students (46.0%) disagreed, suggesting that many perceived group work as time-consuming. In terms of emotional experience, 101 students (47.4%) agreed that group learning was not frustrating or stressful, although 51 students (23.9%) disagreed, reflecting mixed experiences. Furthermore, 120 students (56.3%) agreed that group learning was a very good way to learn block content, indicating overall positive perceptions of the group-based approach.

Table 3 presents the mean and SD of these responses. The highest mean score (3.59) corresponded to the statement “Working in a group was better than working alone,” indicating general agreement. The lowest mean score (2.72) was associated with the statement “Group learning did not take too much of my time,” reflecting a tendency toward disagreement about time efficiency.

**Table 3:** Average perceptions of PBL group effectiveness (process construct) based on the Likert scale (n = 213) PBL, problem-based learning

Process construct	Average	SD
Working in a group was better than working alone	3.59	1.144
When working alone, I did not learn as much as I did when I worked in a group	3.24	1.188
Group learning did not take too much of my time	2.72	1.113
Learning in a group was not frustrating and stressful	3.28	1.030
Group learning was a very good way of learning the content of the block	3.49	1.058

Among the 213 participants, 125 (58.7%) agreed that working in a group was better than working alone, with a corresponding mean score of 3.59 ± 1.144. A total of 80 students (37.4%) agreed that they learned less when working alone than when working in a group, with a mean score of 3.24 ± 1.188. Only 58 participants (27.3%) agreed that group learning did not take too much of their time, reflected in a lower mean score of 2.72 ± 1.113, indicating concerns about the time demands of group work.

In terms of emotional experience, 101 students (47.4%) agreed that learning in a group was not frustrating or stressful, with a mean score of 3.28 ± 1.030. Additionally, 120 participants (56.3%) agreed that group learning was a very good way of learning block content, corresponding to a mean score of 3.49 ± 1.058. Overall, these findings indicate moderately positive perceptions of group work in PBL, particularly in terms of collaboration and content understanding.

Gender-based differences in the process construct

**Figure 3:**
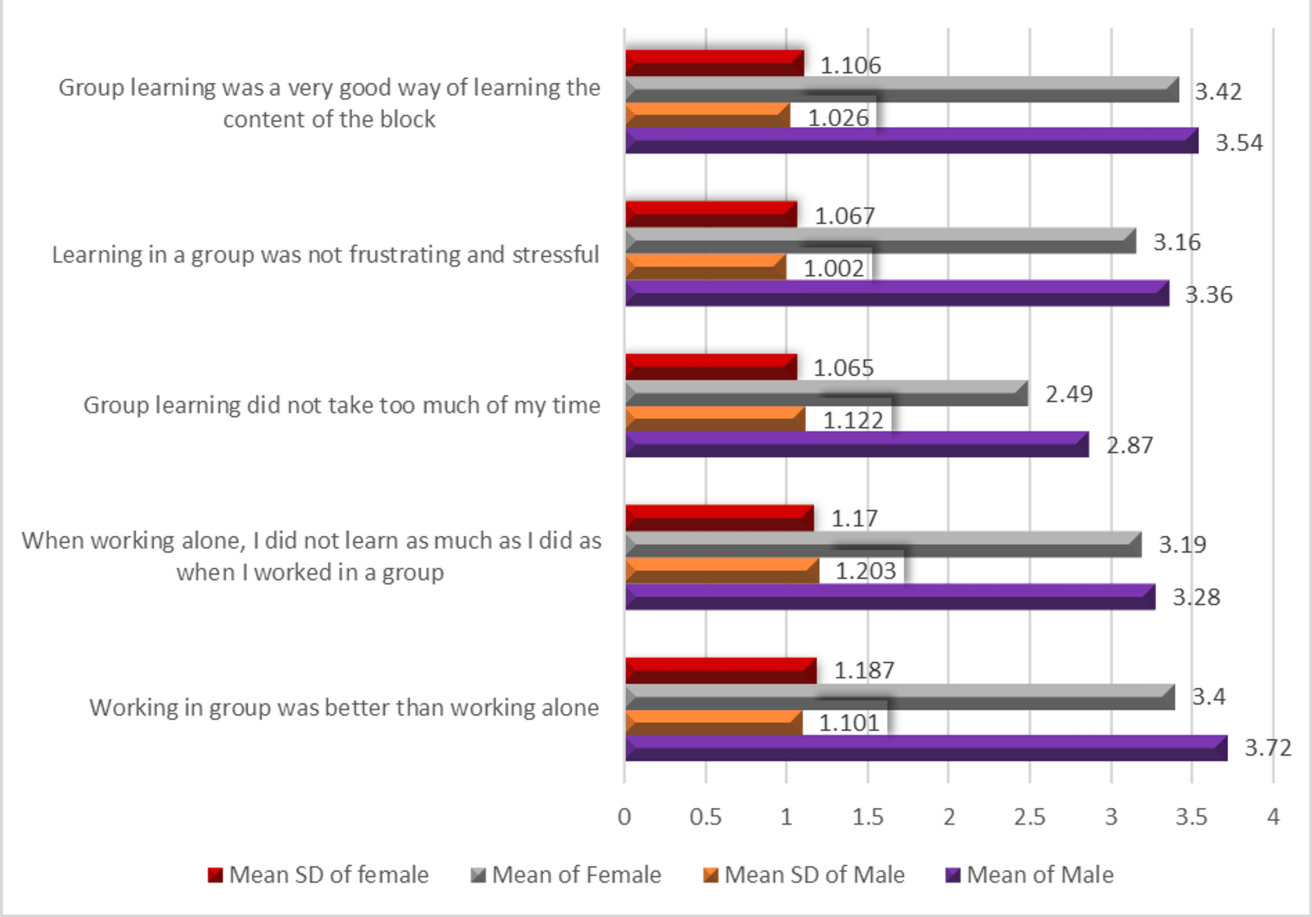
Gender-based differences in average perceptions of PBL group effectiveness (process construct) PBL, problem-based learning

Figure 3 shows the mean scores for male and female students. Male students reported slightly higher mean scores across all process-related items. The highest-rated item for males was “Working in a group was better than working alone” (M = 3.72), while the lowest was “Group learning did not take too much of my time” (M = 2.87). Female students also showed overall agreement with the effectiveness of group work but reported slightly lower scores. Their highest score was “Group learning was a very good way of learning the content” (M = 3.42), while the lowest was “Group learning did not take too much of my time” (M = 2.49), suggesting that female students perceived group work as more time-consuming than males.

Content construct

Table 4 summarizes the percentage distribution of students’ perceptions regarding the content construct. High levels of agreement were reported across all items. A total of 174 students (81.7%) reported benefiting from the input of other group members, while 172 (80.8%) agreed that information was shared freely within the group. Additionally, 166 (77.7%) felt that effective learning took place during group work, and 162 (76.1%) stated that they became more tolerant through this collaborative process. Furthermore, 164 students (77.0%) noted an improvement in their ability to collaborate with peers from diverse backgrounds. However, 141 (66.0%) indicated that group work helped clarify confusing content after lectures, suggesting that while group work was generally beneficial, it did not fully resolve conceptual difficulties for all students.

**Table 4:** Frequency distribution of perceptions on the effectiveness of group work in PBL (content construct) based on the Likert scale (n = 213) PBL, problem-based learning

Content construct	Agree	Neutral	Disagree
I learn to be tolerant in group sessions	76.1%	22.1%	1.9%
I learn to work successfully with students from different social and cultural groups	77.0%	16.9%	6.1%
My group worked together effectively (learning happened)	77.7%	12.2%	11.2%
Members in my group shared information freely	80.8%	8.9%	10.3%
I benefited from the input of other group members	81.7%	13.6%	4.6%
I became more perceptive and sensitive to the needs of others during group work	69.5%	25.4%	5.1%
Group work helped me make sense of some areas of the year's studies that were still confusing after the lectures, tutorials, and/or practicals	66.0%	22.5%	11.5%

Table 5 further supports this trend. The highest mean score was for “I benefited from the input of other group members” (M = 4.11), while the lowest was for “Group work helped me make sense of some areas of the year's studies that were still confusing after the lectures, tutorials, and/or practicals” (M = 3.68).

**Table 5:** Average perceptions of PBL group effectiveness (content construct) based on the Likert scale (n = 213) PBL, problem-based learning

Content construct	Average	SD
I learn to be tolerant in group sessions	3.94	0.705
I learn to work successfully with students from different social and cultural groups	3.94	0.802
My group worked together effectively (learning happened)	4.00	1.086
Members in my group shared information freely	4.08	0.968
I benefited from the input of other group members	4.11	0.894
I became more perceptive and sensitive to the needs of others during group work	3.87	0.853
Group work helped me make sense of some areas of the year's studies that were still confusing after the lectures, tutorials, and/or practicals	3.68	1.016

Gender-based differences in the content construct

**Figure 4:**
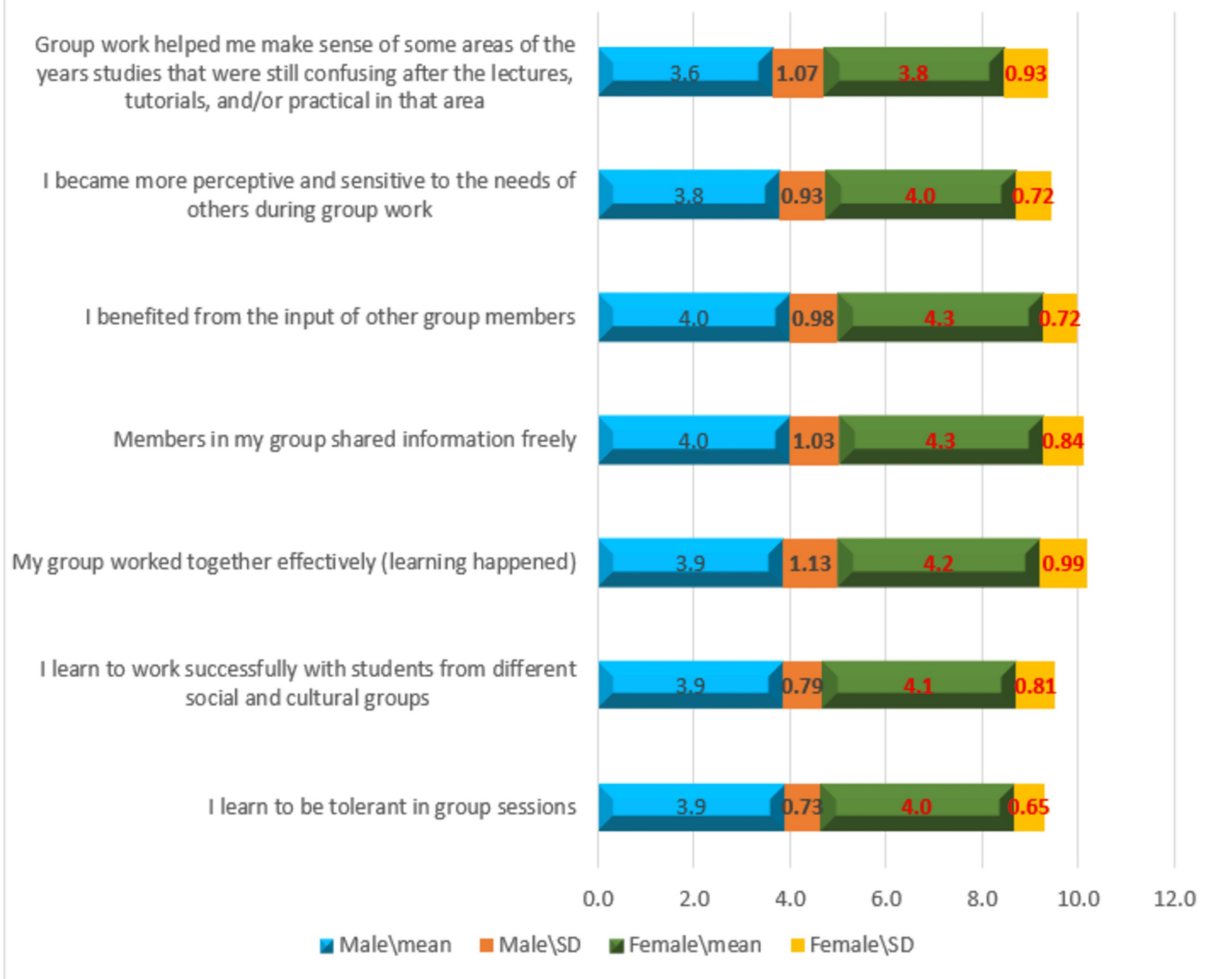
Gender-based average perceptions of PBL group effectiveness (content construct) based on the Likert scale PBL, problem-based learning

Figure 4 presents the detailed mean scores for the content construct of PBL group effectiveness by gender. Female students consistently reported higher mean scores than males across all content-related items. Their strongest agreement was with “I benefited from the input of group members” (M = 4.29, SD = 0.721). They also scored higher in perceived tolerance, communication, and interpersonal sensitivity. Male students, while also reporting generally positive perceptions, had slightly lower average scores. Their highest-rated item was “I benefited from the input of group members” (M = 3.99). This pattern suggests that female students may perceive greater socioemotional and educational benefits from PBL group work.

Inferential statistical analysis

A series of one-way ANOVA tests was conducted to examine the influence of gender and academic level on students’ perceptions of group effectiveness in PBL, focusing on both the process and content constructs.

With respect to gender, a one-way ANOVA revealed a statistically significant effect on perceptions of the process construct, F(1, 211) = 3.942, p = 0.048. Male and female students differed significantly in their views, with males rating the group process dimension slightly more positively. Gender also had a significant impact on the content construct, F(1, 211) = 7.632, p = 0.006, with female students showing more favorable perceptions of content-related aspects of group effectiveness compared to their male counterparts. These results suggest that gender influences how students perceive the value of group work across both procedural and academic dimensions.

Regarding academic level, a one-way ANOVA showed a significant effect on perceptions of the process construct, F(2, 210) = 6.532, p = 0.002. Post hoc Tukey’s honestly significant difference analysis indicated that third-year students reported significantly higher ratings than first-year students (mean difference = 0.467, p = 0.001). However, differences between first- and second-year students (p = 0.057) and between second- and third-year students (p = 0.411) were not statistically significant. A similar trend was observed for the content construct, where ANOVA results revealed significant differences across academic levels. Specifically, third-year students rated content-related group effectiveness more favorably than first-year students (mean difference = 0.288, p = 0.012), while no significant differences were found between first- and second-year (p = 0.198) or second- and third-year students (p = 0.471).

## Discussion

This study explored undergraduate medical students’ perceptions of group effectiveness in PBL at Qassim University using both quantitative and comparative approaches. Two critical constructs, process and content, were used to evaluate students’ experiences, alongside demographic factors such as gender and academic level. The findings reinforce the well-documented pedagogical value of PBL.

Overall, students demonstrated generally positive attitudes toward group learning, consistent with previous research in Saudi Arabia and internationally [1,2,6,7]. A majority of students (125; 58.7%) preferred collaborative learning, agreeing that working in groups was more effective than working individually. This supports the recognized educational value of group interactions in enhancing understanding and critical thinking within PBL environments [3,4].

However, mixed perceptions emerged regarding the time efficiency of group learning. While many students acknowledged its benefits, nearly half (98; 46%) reported that group activities were time-consuming. Such concerns align with prior studies highlighting that PBL often demands substantial time investment, which can cause frustration and potentially reduce student satisfaction [9,16]. These challenges underscore the importance of effective time management strategies and proactive facilitator involvement to optimize group performance and minimize student stress [12,16].

The content construct of group effectiveness, which included knowledge sharing, peer contribution, and socioemotional learning, received particularly strong positive ratings. Most students recognized the value of peer input and appreciated the development of tolerance, communication, and interpersonal sensitivity skills. These competencies are essential for both professional and personal development, aligning with modern medical education goals to foster collaborative skills [14,20,22]. Nevertheless, a somewhat lower level of agreement regarding group work’s ability to clarify confusing lecture content suggests that group discussions alone may not fully resolve conceptual difficulties, indicating a potential need for supplementary instructional support alongside PBL [11].

Gender-based differences and academic level effects

Significant gender differences were observed in perceptions of group effectiveness. Male students rated the process construct, including aspects such as group dynamics and efficiency, slightly more positively than female students (F(1, 211) = 3.942, p = 0.048). In contrast, female students rated the content construct significantly higher, reflecting a stronger appreciation for the educational and socioemotional benefits of group interactions (F(1, 211) = 7.632, p = 0.006). Females also expressed greater agreement regarding peer contributions and valued tolerance, communication, and interpersonal sensitivity more than males.

These findings align with existing literature, which suggests that female medical students often place greater value on collaborative learning and group cohesion, enhancing both engagement and perceived benefits in PBL settings [6,7,8,20]. Their higher social sensitivity and empathy levels may explain these results, highlighting the importance of gender-sensitive approaches in medical education [20,22].

Academic progression also significantly influenced perceptions. Third-year students expressed more favorable views of both process and content constructs compared to first-year students (process: mean difference = 0.467, p = 0.001; content: mean difference = 0.288, p = 0.012). This trend likely reflects increased familiarity with PBL, improved self-directed learning skills, and greater comfort with collaborative problem-solving as students advance through their training. These findings are consistent with research from other Saudi medical schools, which shows that students’ attitudes toward PBL generally improve with curricular progression [2,13,14].

First-year students may initially find group work challenging, particularly in managing time and adapting to student-centered learning approaches. This transition can negatively affect early perceptions of PBL [16]. Therefore, targeted support during the initial stages, such as teamwork training, communication workshops, and self-regulation strategies, could promote more positive and productive group experiences from the outset [16,18].

Implications for medical education

The observed gender and academic-level differences highlight the need for tailored educational strategies within PBL curricula. Facilitators should adopt gender-sensitive approaches to optimize group dynamics, ensuring that both male and female students benefit equally from collaborative learning. Additionally, scaffolding curricula to gradually develop group work skills and PBL competencies may enhance engagement, satisfaction, and learning outcomes across all levels [6,9,16].

Addressing time management concerns and supplementing group discussions with complementary instructional methods could further mitigate challenges reported by students. Facilitators play a central role in guiding groups to balance thorough exploration of topics with efficient use of time, creating a supportive environment that maximizes the benefits of PBL [12,16].

Limitations

Several limitations should be acknowledged. First, the cross-sectional design captures perceptions at a single point in time, limiting the ability to infer causality or track changes longitudinally. Second, the medical curriculum at Qassim University follows a hybrid model, integrating traditional lectures, PBL, and self-directed learning, which may influence student perceptions and limit generalizability to purely PBL-based curricula. Finally, although the study was conducted during the COVID-19 pandemic, data collection was not adversely affected. Regular online meetings with students facilitated communication, ensured adherence to safety protocols, and contributed to a satisfactory response rate.

This study will aid curriculum developers in enhancing teaching quality and sustaining Qassim University’s leadership in innovative education, while also benefiting other universities. It aligns with Vision 2030’s goal of improving higher education quality and supports evidence-based teaching reforms in medical colleges across the Kingdom. By addressing a local knowledge gap and providing context-specific insights, the study can also contribute to broader international discussions on culturally adaptable and effective PBL strategies.

## Conclusions

This study adds to the growing evidence supporting the effectiveness of PBL in medical education, highlighting the positive perceptions students hold toward group work. It further elucidates important nuances related to gender and academic level, suggesting the value of personalized educational interventions. By addressing these factors, medical schools can optimize PBL experiences, promote deeper learning, and better prepare students for collaborative clinical practice.

## Appendices

Appendix A 

**Table 6:** Questionnaire assessing students’ perceptions of small-group PBL sessions and the effectiveness of learning in these sessions PBL, problem-based learning

					First section: Personal data:
					Age
Male ( ) Female ( )					Sex
Level 1 Level 2 Level 3					Level
					Second section: Processes construct:
Strongly disagree	Disagree	Natural	Agree	Strongly agree	
					Working in a group was better than working alone
					When working alone, I did not learn as much as I did when I worked in a group
					Group learning did not take too much of my time
					Learning in a group was not frustrating and stressful
					Group learning was a very good way of learning the content of the block
					Section three: Content construct:
Strongly disagree	Disagree	Natural	Agree	Strongly agree	
					I learn to be tolerant in group sessions
					I learn to work successfully with students from different social and cultural groups
					My group worked together effectively (learning happened)
					Members in my group shared information freely
					I benefited from the input of other group members
					I became more perceptive and sensitive to the needs of others during group work
					Group work helped me make sense of some areas of the years studies that were still confusing after the lectures, tutorials, and/or practical’s in that area
